# Efficacy of Intravenous Lidocaine During Endoscopic Submucosal Dissection for Gastric Neoplasm: A Randomized, Double-Blind, Controlled Study

**DOI:** 10.1097/MD.0000000000003593

**Published:** 2016-05-06

**Authors:** Ji Eun Kim, Jong Bum Choi, Bon-Nyeo Koo, Hae Won Jeong, Byung Ho Lee, So Yeon Kim

**Affiliations:** From the Department of Anesthesiology and Pain Medicine, Ajou University School of Medicine, Suwon (JEK, JBC, HWJ, BHL), and Department of Anesthesiology and Pain Medicine, Severance Hospital, Anesthesia and Pain Research Institute, Yonsei University College of Medicine, Seoul (BNK, SYK), Republic of Korea.

## Abstract

Endoscopic submucosal dissection (ESD) is an advanced therapy for early gastric neoplasm and requires sedation with adequate analgesia. Lidocaine is a short-acting local anesthetic, and intravenous lidocaine has been shown to have analgesic efficacy in surgical settings. The aim of this study was to assess the effects of intravenous lidocaine on analgesic and sedative requirements for ESD and pain after ESD.

Sixty-six patients scheduled for ESD randomly received either intravenous lidocaine as a bolus of 1.5 mg/kg before sedation, followed by continuous infusion at a rate of 2 mg/kg/h during sedation (lidocaine group; n = 33) or the same bolus and infusion volumes of normal saline (control group; n = 33). Sedation was achieved with propofol and fentanyl. The primary outcome was fentanyl requirement during ESD. We recorded hemodynamics and any events during ESD and evaluated post-ESD epigastric and throat pain.

Fentanyl requirement during ESD reduced by 24% in the lidocaine group compared with the control group (105 ± 28 vs. 138 ± 37 μg, mean ± SD; *P* < 0.001). The lidocaine group reached sedation faster [40 (20–100) vs. 55 (30–120) s, median (range); *P* = 0.001], and incidence of patient movement during ESD decreased in the lidocaine group (3% vs. 26%, *P* = 0.026). Numerical rating scale for epigastric pain was significantly lower at 6 hours after ESD [2 (0–6) vs. 3 (0–8), median (range); *P* = 0.023] and incidence of throat pain was significantly lower in the lidocaine group (27% vs. 65%, *P* = 0.003). No adverse events associated with lidocaine were discovered.

Administration of intravenous lidocaine reduced fentanyl requirement and decreased patient movement during ESD. Moreover, it alleviated epigastric and throat pain after ESD. Thus, we conclude that the use of intravenous adjuvant lidocaine is a new and safe sedative method during ESD.

## INTRODUCTION

Endoscopic submucosal dissection (ESD) is the current standard therapy for premalignant lesions and early stomach cancers because this procedure markedly improves en bloc resection, histologically complete resection, and localized tumor recurrence rates compared with endoscopic mucosal dissection.^1^ However, despite the many advantages of ESD, caution is required to achieve adequate sedation because of the technical difficulties and long procedure duration. As ESD outcomes and complications can be determined by sedation method,^2,3^ it is important to provide better sedation during ESD. Furthermore, efforts to reduce post-ESD pain are needed because moderate to severe epigastric pain is common after ESD.^4^ Several studies have reported methods for reducing pain from ESD, including transdermal fentanyl patch, submucosal injection of local lidocaine or bupivacaine, intravenous (IV) dexamethasone, and IV magnesium.^5–9^

Lidocaine, developed in 1948, is an amide-type short-acting local anesthetic and has analgesic, antihyperalgesic, and anti-inflammatory properties.^10–12^ IV lidocaine was originally intended to be an antiarrhythmic drug but has been used in various surgical settings.^10–12^ Perioperative use of IV lidocaine, especially for abdominal surgery, has been shown to reduce anesthetic or analgesic requirements, postoperative pain, postoperative nausea and vomiting, and has resulted in earlier return of bowel function and shorter hospital stay.^10–14^ We previously reported that use of IV magnesium, which showed analgesic efficacy in surgical settings, also reduced the analgesic requirement during ESD and decreased epigastric pain after ESD.^9^ In accordance, IV lidocaine might be another effective analgesic for ESD.

This randomized study assessed the effects of IV lidocaine administered as a bolus injection (1.5 mg/kg) followed by a continuous infusion (2 mg/kg/h) on sedative and analgesic requirements during ESD and on post-ESD pain.

## METHODS

This study was approved by the institutional review board and Hospital Research Ethics Committee of Severance Hospital in the Yonsei University Health System (protocol number: 4-2014-0933) and was registered at http://clinicaltrials.gov (registration number: NCT02543411). Between September 2015 and November 2015, 66 patients aged between 20 and 80 years who were scheduled for ESD to treat early gastric neoplasm (pathologically diagnosed gastric adenoma or cancer) were included. ESD was scheduled on the basis of standard criteria: histological diagnosis of a well or moderately differentiated adenocarcinoma or dysplasia (adenoma); tumor invasion of the mucosa or minute submucosal layers defined by endoscopic ultrasonography; tumor size ≤3 cm if minute submucosal invasion or tumor with ulceration was suspected; and tumor of any size if it was a differentiated adenocarcinoma without ulceration or submucosal invasion. Written informed consent was obtained from all patients before randomization. Patients were excluded if they met at least one of the following criteria: hypersensitivity to lidocaine, chronic pain, chronic abuse of opioid or nonsteroidal anti-inflammatory drug, atrioventricular block, liver or renal dysfunction, multiple gastric lesions, or gastrointestinal pain.

### Interventions

Patients were randomly assigned into either the lidocaine group or the control group in a 1 : 1 ratio by computer-generated randomization (http://www.random.org). Study medications were prepared in bags and syringes of identical appearance by a staff nurse who was not involved in the study. Lidocaine was prepared as follows: 1.5 mg/kg of 1% lidocaine diluted with normal saline to a total volume of 100 mL for the bolus dose and 20 mL of 1% lidocaine mixed with 20 mL of normal saline for a concentration of 5 mg/mL for continuous infusion. Patients in the lidocaine group (n = 33) received IV lidocaine as a bolus dose of 1.5 mg/kg before sedation, followed by a continuous infusion at a rate of 2 mg/kg/h during sedation. Patients in the control group (n = 33) received an equal volume of normal saline as a placebo following the same protocol used for lidocaine bolus and infusion. The bolus was administered over 10 minutes before sedation induction at the pre-anesthetic care unit, and continuous infusion was started simultaneously with sedation induction and continued throughout the procedure. The anesthetic administrator, endoscopists, nurses, patients, outcome assessors, and data analysts were all blinded to patient assignment until analysis completion.

All patients were sedated using Monitored Anesthesia Care performed by an experienced anesthesiologist. During the procedure, pulse oxygen saturation (SpO_2_), noninvasive blood pressure (NIBP), capnography, and electrocardiography (ECG) with respiratory activity via thoracic leads were monitored. Supplemental oxygen was provided via the nasal cannula at a rate of 3 L/min. After loading 5 to 7 mL/kg of normal saline or Ringer's lactate solution, fluid infusion was maintained at a rate of 3 to 5 mL/kg/h. Before the procedure, all patients were informed to indicate any discomfort or pain during procedure by raising his or her right hand.

All patients received butylscopolamine (Buscopan; Handok Pharm Co. Ltd., Cheongju, Korea) as premedication. The patients were initially sedated with an IV bolus of 0.5 mg/kg of propofol (Fresofol MCT 1%; Fresenius Kabi, Graz, Austria) and 1 μg/kg of fentanyl (Hana Pharm, Seoul, Korea). Sedation was maintained with continuous propofol infusion at a rate of 60 μg/kg/min, along with an intermittent fentanyl bolus of 0.5 μg/kg. Propofol infusion was terminated upon coagulation, which began after specimen dissection. The targeted depth of sedation was 3 or 4 of the Modified Observer Assessment of Alertness/Sedation (MOAA/S) scale. MOAA/S scores range from 0 to 6 (0 = unresponsive to deep stimuli; 1 = unresponsive to shaking; 2 = responsive to shaking only; 3 = responsive to loud verbal commands; 4 = lethargic response to normal verbal command; 5 = responsive and alert; and 6 = agitated).^15^ In cases with MOAA/S ≥5 or when deeper sedation was requested by the endoscopist, an additional 10 mg of propofol was provided as a bolus injection, and the propofol infusion rate was increased stepwise by 10 μg/kg/min. If heart rate (HR) or mean blood pressure (MBP) increased by >20% of baseline or if the patient raised his or her hand to indicate pain according to the prior instructions, a 0.5 μg/k g bolus of fentanyl was administered. If an increase of MBP persisted despite propofol or fentanyl administration, 10 mg of IV labetalol was administered. In cases of MBP <60 mm Hg or HR <40 beats/min, IV ephedrine 4 mg or atropine 0.5 mg was administered, respectively. In apnea or desaturation with SpO_2_ <90%, supplemental oxygen was increased to 5 L/min, and airway modification, including chin lift, jaw thrust, or assisted mask ventilation, was applied on the basis of the clinical judgment of the attending anesthesiologist. If desaturation persisted despite such interventions, the propofol infusion was decreased or stopped.

ESD procedures were performed by experienced endoscopists who were blinded to patient assignment, and a single-channel upper gastrointestinal endoscope (GIF-Q260J or GIF-H260Z; Olympus Optical Co. Ltd., Tokyo, Japan) was used. The procedure was performed in the following sequence: the resection margin was marked, the mucosa was elevated, the submucosa was incised/dissected, and the resection bed was coagulated. After identification of the target lesion, several dots were made circumferentially 5 mm outside of the tumor, using argon plasma coagulation. Then, submucosal injection of a saline solution and an epinephrine mixture (0.1% epinephrine 1 mL and 0.9% normal saline 9 mL and 8% indigo carmine) was performed to elevate the lesion off of the muscular layer. A circumferential incision was made in the mucosa with a needle knife in order to separate the lesion from the surrounding non-neoplastic mucosa. Next, the submucosal layer was dissected directly using an insulated-tip knife, until complete removal was achieved. If necessary, submucosal injections were repeated during the procedure, and endoscopic hemostasis and ablation of visible vessels were achieved using a hemoclip or hemostatic forceps. After the procedure, patients were transferred to a recovery room and monitored with regard to SpO_2_, NIBP, and ECG. Patients were assessed by nursing staff using Aldrete scores and discharged when they reached a total Aldrete score of 8, including a respiratory score of 2.^15^

### Data Collection

Total administered doses of fentanyl and propofol during ESD were recorded. HR and MBP were collected at 7 separate time points: before sedation induction (T0); after sedation induction (T1); submucosal injection of epinephrine (T2); 5, 10, and 20 minutes after submucosal dissection (T3, T4, and T5); end of the procedure (T6). The degree of bleeding (minimal, moderate, severe) and the presence of fibrosis was checked during the procedure. The definition of “en bloc resection” was tumor resection in a single piece without fragmentation and “complete resection” was complete tumor resection with a histologically tumor-negative resection margin. “Procedure time” meant the time from endoscopic insertion to the endoscopic removal and “time to sedation” meant the time from propofol bolus injection to moderate-to-deep sedation (MOAA/S score ≤3). Gagging during endoscopic insertion and involuntary movement during the procedure, such as raising of the head, moving of hands toward the endoscopy site, attempting to sit in the upright position, and retching, were recorded.

Upon arrival in the recovery room, MOAA/S scale was assessed for each patient. Epigastric and throat pain were checked in the recovery room or in the ward at 30 minutes, 6, and 24 hours after the procedure. The pain intensity was checked with an 11-point numerical rating scale (NRS; no pain = 0, worst possible pain = 10). When patients reported an NRS score >5 or patients requested additional analgesics, 0.5 μg/kg of IV fentanyl was administered in the recovery room, and 30 mg of IV ketorolac or 50 mg of IV tramadol was administered in the ward. Nausea or vomiting was recorded up to 24 hours after the procedure. The length of recovery room stay and hospital stay were recorded. Adverse events related to the procedure such as leukocytosis (>11,000/μL), fever (>38°C), bleeding (required blood transfusion or >2 g/dL decrease in hemoglobin level after ESD), perforation (direct endoscopic visualization of mesenteric fat or evidence of free air on abdominal x-ray), atelectasis, or pneumonia (in post-procedural chest x-ray), as well as events related to lidocaine administration such as arrhythmias or signs of lidocaine toxicity, were assessed throughout the hospital stay.

### Statistical Analysis

The primary endpoint of this study was total administered dose of fentanyl during ESD. In a previous study, the mean ± standard deviation (SD) of administered fentanyl dose during ESD was 126 ± 41 μg.^9^ When a 25% reduction of fentanyl consumption is considered to be clinically relevant,^16^ an enrollment of 28 subjects per each group was required at a significance level of 5% and a power level of 80%. For possible dropouts, 33 patients per each group were included.

Data are expressed as mean ± SD, median (range), or number of patients (proportion). Normality of distribution was assessed with a Q–Q plot and the Shapiro–Wilk test. The independent *t* test and the Mann–Whitney *U* test were used for parametric and nonparametric data, respectively. The Chi-square test or Fisher exact test was used for categorical variables as appropriate. Repeated-measured data within the group, such as HR and MBP, were analyzed with a linear mixed model. When the interaction was statistically significant, posthoc test was performed and the *P* value was adjusted with Bonferroni correction. A *P* value of <0.05 was considered statistically significant. Statistical analysis was performed using PASW Statistics 20 (SPSS Inc., Chicago, IL) and SAS 9.2 (SAS Inc., Cary, NC).

## RESULTS

**FIGURE 1:**
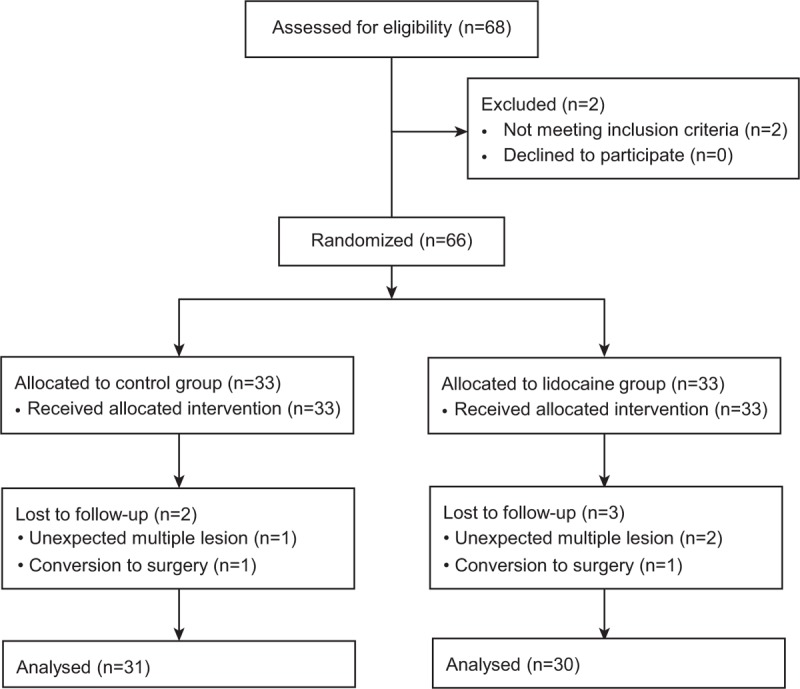
Patient assignment to study groups and treatment protocols.

Of 68 patients assessed for eligibility, 66 patients were enrolled and randomly assigned to the study groups; 61 (92%) completed the study (Figure 1). Five patients (2 in the control group and 3 in the lidocaine group) were eliminated from the analysis for the following reasons: unexpected multiple lesions (n = 3) and conversion to surgery (n = 2). There were no significant differences between the 2 groups with regard to patient characteristics and procedure details, except for snoring history (Table 1).

**TABLE 1:**
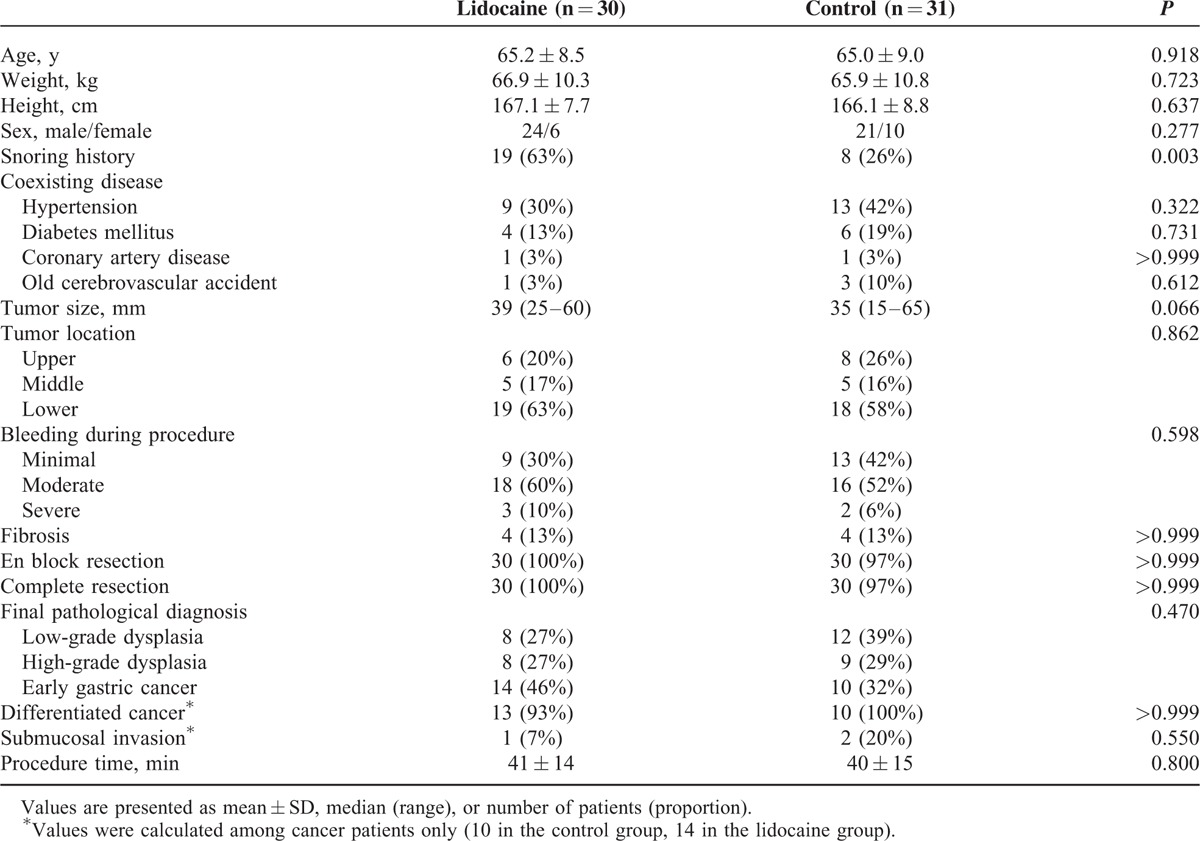
Patient Characteristics and Procedure Details

### Sedation and Recovery Profile

**FIGURE 2:**
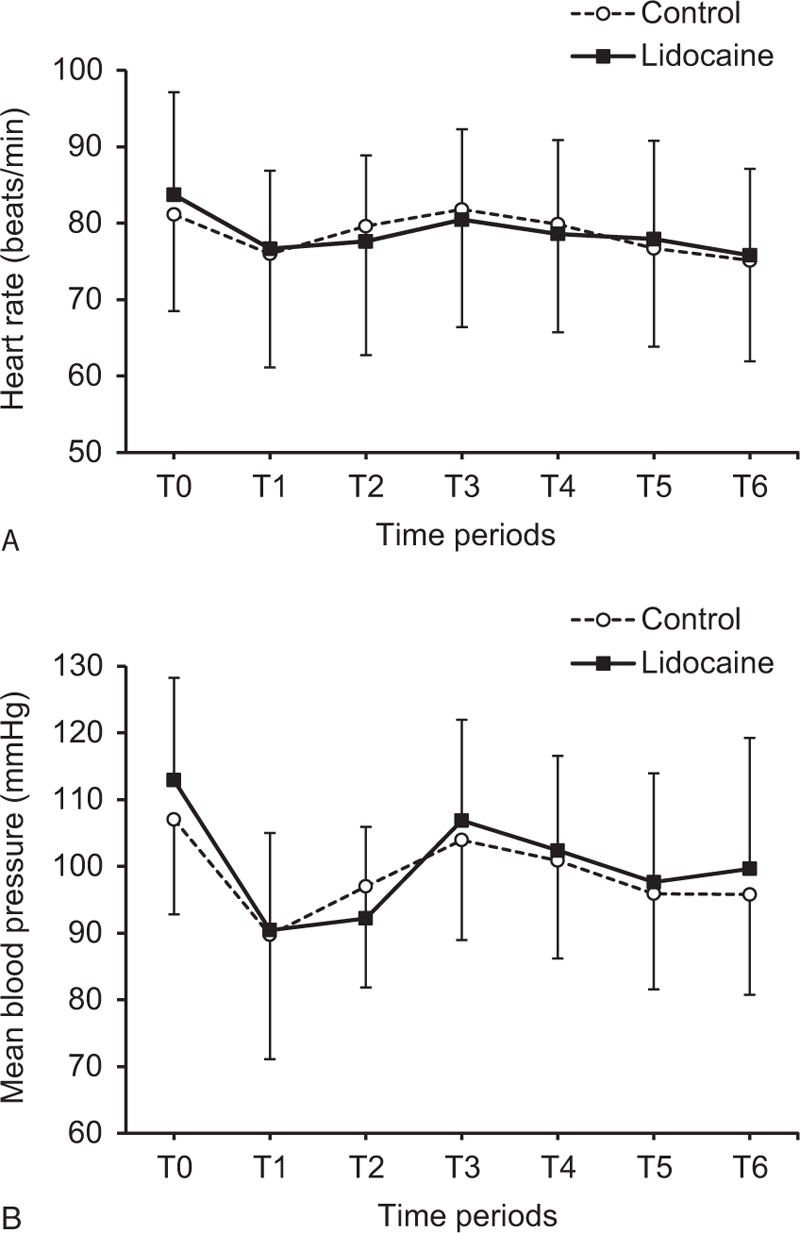
Changes in (A) heart rate and (B) mean blood pressure during endoscopic submucosal dissection. T0, before sedation induction; T1, after sedation induction; T2, submucosal injection of epinephrine; T3, 5 minutes after submucosal dissection; T4, 10 minutes after submucosal dissection; T5, 20 minutes after submucosal dissection; T6, end of the procedure. Data are expressed as mean ± SD.

Data related to events and drug administration during the procedure are presented in Table 2. After administration of propofol and fentanyl boluses, the lidocaine group was sedated faster than the control group [40 (20–100) vs. 55 (30–120) s, median (range); *P* = 0.001]. The total administered dose of fentanyl during the procedure was 24% lower in the lidocaine group than in the control group (105 ± 28 vs. 138 ± 37 μg, mean ± SD; *P* < 0.001). The total administered dose of propofol was 18% lower in the lidocaine group, although this result was not significant (*P* = 0.085). In addition, the incidence of patient involuntary movement during the procedure was lower in the lidocaine group (3% vs. 26%, *P* = 0.026). Hemodynamics during the procedure was similar between the 2 groups (Figure 2).

**TABLE 2:**
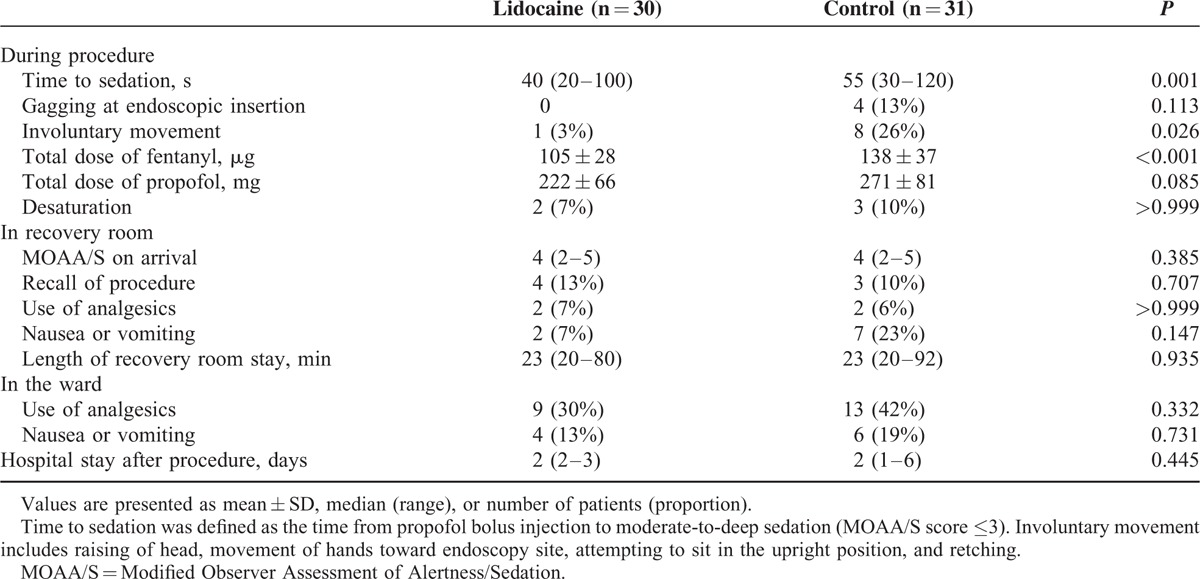
Data Related to Sedation and Recovery

Data related to events and drug administration after the procedure are presented in Table 2. MOAA/S score on arrival to the recovery room and length of stay in the recovery room were similar between the groups. Patient need for additional analgesics and incidence of nausea or vomiting in the recovery room or ward were similar between the groups. Even though incidence of epigastric pain was not different between the groups, NRS score at 6 hours after the procedure was significantly lower in the lidocaine group [2 (0–6) vs. 3 (0–8), median (range); *P* = 0.023] (Table 3). Moreover, fewer patients in the lidocaine group complained of throat pain (27% vs. 65%, *P* = 0.003) (Table 3).

**TABLE 3:**
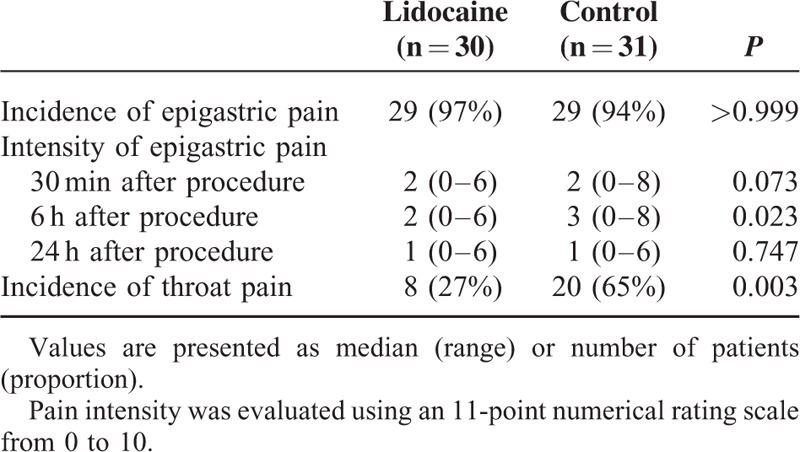
Epigastric Pain and Throat Pain During 24 hours After the Procedure

No cardiovascular or neurological side effects associated with IV lidocaine were discovered in any patients of this study.

### Complications

Complications after the procedure were similar between the 2 groups, and all complications resolved before hospital discharge (Table 4).

**TABLE 4:**
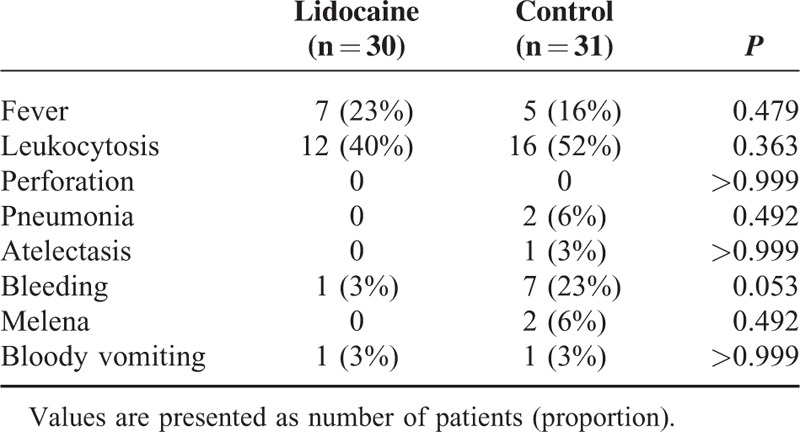
Complications Related to the Procedure

## DISCUSSION

In this randomized, double-blind, placebo-controlled trial, IV lidocaine administered as a bolus of 1.5 mg/kg and then as a continuous infusion of 2 mg/kg/h during ESD reduced the fentanyl requirement by 24% and was associated with fewer involuntary movements during ESD. Lidocaine administration also shortened the time to sedation and reduced the severity of epigastric pain as well as the incidence of throat pain after ESD.

IV lidocaine has been used for relieving chronic pain, such as from cancer and neuropathy.^17,18^ It has also shown effectiveness in controlling acute pain, such as postoperative pain.^10–12^ The analgesic mechanisms of IV lidocaine are multifactorial and include a sodium channel blockade (inhibition of spontaneous and evoked neuronal activity) and selective depression of pain transmission through the spinal cord (reduction of postsynaptic depolarization mediated by N-methyl-D-aspartate and neurokinin receptors).^19^ The analgesic efficacy of IV lidocaine has been observed mostly in abdominal surgeries, including colectomy, gastrectomy, and cholecystectomy, with no evidence of efficacy during other surgeries, such as including tonsillectomy, total hip arthroplasty, or cardiac surgery.^10–12,20^ On the basis of these results, the analgesic effects of lidocaine are more potent for visceral pain than somatic pain. Pain during ESD is induced by overextension of the gastric wall and heat coagulation of the muscularis and post-ESD pain is caused by an inflamed artificial gastric ulcer and exposure to gastric acid.^4,21^ Therefore, we hypothesized that IV lidocaine would be beneficial for controlling visceral pain from ESD. As we predicted, administration of IV lidocaine during ESD resulted in less fentanyl consumption during ESD and reduction of epigastric pain intensity after ESD. The half-life of lidocaine is 1.5 to 2 hours following bolus injection or continuous infusion lasting less than 12 hours.^10,22^ Because of the short half-life of IV lidocaine, greater reduction in pain score has been reported in the earlier postoperative period when infusion was discontinued at the end of surgery.^10^ This may contribute to the reason why analgesic effect was noted at 6�hours but not at 24�hours in our results.

Fentanyl is the drug of choice for endoscopy because of its rapid onset, its minimal effect on the cardiovascular system, and its fast recovery time.^15,23^ It is known that the addition of fentanyl can reduce the total dose of propofol, and consequently decrease the risk of rapid, irreversible oversedation, and respiratory events compared with propofol monosedation.^15,23,24^ However, fentanyl itself is also associated with a high risk of respiratory depression, which occurs in a dose-dependent manner and persists longer than the analgesic effect.^25^ Thus, fentanyl-sparing effect of IV lidocaine is meaningful for maintenance of the airway and spontaneous breathing in sedated patients. We found that desaturation incidence during ESD was similar between the 2 groups, even though more patients in the lidocaine group had a snoring history, which is an independent risk factor of respiratory events.^24^ In addition to its fentanyl-sparing effect, IV lidocaine has been shown to have a propofol-sparing effect during general anesthesia; this effect is mediated by an antinociceptive action rather than a hypnotic effect.^26^ Although not statistically significant, we found that the total propofol dose was 18% lower in the lidocaine group (*P* = 0.085), which is consistent with a previous report that lidocaine reduced the propofol requirement by 15% during thyroidectomy.^26^

Addition of IV lidocaine was associated with significantly lower incidence of involuntary movements during ESD in this study. Patient movements usually interrupt the ESD process, which can lead to longer procedure time and possibly result in serious complications, such as bleeding or perforation. This lower incidence of involuntary movements may be due to a more consistent sedation level among the lidocaine group, even though the sedation depth was targeted at 3 or 4 on the MOAA/S scale for both groups. The American Gastroenterological Association recommends use of an adjunctive agent in combination with conventional sedation drugs for difficult-to-sedate patients.^15^ Because administration of IV lidocaine as an adjunctive agent during ESD reduced the incidence of patient movements with less fentanyl consumption, it should be considered as an effective adjunctive agent for such patients.

IV lidocaine reduced the incidence of throat pain from 65% to 27% in our study. A previous study found that IV lidocaine was effective in reducing postoperative sore throat caused by tracheal intubation.^27^ Although no prior studies reported throat pain after ESD, there is evidence that oropharyngeal mucosal injury is common during insertion of a transesophageal echocardiography probe (55%).^28^ Thus, we speculated that similar injuries can occur during insertion of a gastrointestinal endoscopy probe. Furthermore, to-and-fro movement of the endoscopy probe throughout the procedure may further worsen the injury. As sore throat is the source of dissatisfaction and discomfort after a procedure, more caution is needed during the manipulation of an endoscopy probe, and efforts to reduce throat pain with drugs should be taken.

We did not observe any cardiovascular or neurological side effects associated with IV lidocaine. The clinically effective pain-relieving concentration of serum lidocaine is estimated to be 2 to 10 μg/mL, which is sufficient to reduce discharge of the peripheral pain-transducing nerves, A-δ, and C-fibers.^29^ Toxic concentration of lidocaine (>5 μg/mL) has been reported after longer-duration lidocaine infusion during cardiac or abdominal surgery.^12^ However, a meta-analysis concluded that the incidence of adverse cardiovascular or neurological events did not increase with IV lidocaine infusion.^12^ A previous study showed that IV lidocaine as a bolus injection of 1.5 mg/kg at induction of anesthesia followed by a continuous infusion of 2 mg/kg/h during laparoscopic colectomy resulted in a plasma concentration of 2.7 ± 1.1 μg/mL by the end of surgery.^14^ In that study, bolus dose and infusion rate of lidocaine were the same as in our study. However, their anesthesia duration was longer (169 ± 47 minutes) than our procedure time (41 ± 14 minutes), leading us to assume that the plasma concentration of lidocaine in our patients was ≤2.7 ± 1.1 μg/mL.

A limitation of this study is the small number of patients, which restricts our ability to assess the safety of IV lidocaine for ESD. As our sample size calculation was based on the total administered dose of fentanyl during ESD, a larger-scale trial is needed to determine whether some patients with coexisting disease are more likely to develop toxicity.

In conclusion, IV lidocaine administered as a bolus of 1.5 mg/kg and then as a continuous infusion of 2 mg/kg/h reduced fentanyl requirements and incidence of patient movement during ESD. It also lowered the severity of epigastric pain and the incidence of throat pain after ESD. As these beneficial effects were not associated with any adverse effects, IV lidocaine as an adjuvant with conventional sedation drugs should be considered as a new component of sedative medications for ESD.
